# Somatosensory Stimulation With XNKQ Acupuncture Modulates Functional Connectivity of Motor Areas

**DOI:** 10.3389/fnins.2019.00147

**Published:** 2019-03-11

**Authors:** Till Nierhaus, Yinghui Chang, Bin Liu, Xuemin Shi, Ming Yi, Claudia M. Witt, Daniel Pach

**Affiliations:** ^1^Neurocomputation and Neuroimaging Unit, Department of Education and Psychology, Freie Universität Berlin, Berlin, Germany; ^2^Department of Neurology, Max Planck Institute for Human Cognitive and Brain Sciences, Leipzig, Germany; ^3^First Teaching Hospital of Tianjin University of Traditional Chinese Medicine, Tianjin, China; ^4^Charité-Universitätsmedizin Berlin, Corporate Member of Freie Universität Berlin, Humboldt-Universität zu Berlin, Berlin Institute of Health, Institute for Social Medicine, Epidemiology and Health Economics, Berlin, Germany; ^5^Neuroscience Research Institute and Department of Neurobiology, School of Basic Medical Sciences, Peking University, Beijing, China; ^6^Key Laboratory for Neuroscience, Ministry of Education/National Health Commission, Peking University, Beijing, China; ^7^Institute for Complementary and Integrative Medicine, University of Zurich and University Hospital Zurich, Zurich, Switzerland

**Keywords:** resting-state fMRI, acupuncture, functional connectivity, centrality, stroke, red nucleus, precuneus

## Abstract

Xingnao Kaiqiao (XNKQ) acupuncture is an acupuncture technique used for stroke patients. In 24 healthy volunteers, we applied this complex acupuncture intervention, which consists of a manual needle-stimulation on five acupuncture points (DU26 unilaterally, PC6, and SP6 bilaterally). XNKQ was compared to three control conditions: (1) insertion of needles on the XNKQ acupuncture points without stimulation, (2) manual needle-stimulation on five nearby non-acupuncture points, and (3) insertion of needles on the non-acupuncture points without stimulation. In a within-subject design, we investigated functional connectivity changes in resting-state functional magnetic resonance imaging (fMRI) by means of the data-driven eigenvector centrality (EC) approach. With a 2 × 2 factorial within-subjects design with two-factor stimulation (stimulation vs. non-stimulation) and location (acupuncture points vs. non-acupuncture points), we found decreased EC in the precuneus after needle-stimulation (stimulation<non-stimulation), whereas the factor location showed no statistically significant EC differences. XNKQ acupuncture compared with needle-stimulation on non-acupuncture points showed decreased EC primarily in subcortical structures such as the caudate nucleus, subthalamic nucleus, and red nucleus. *Post-hoc* seed-based analysis revealed that the decrease in EC was mainly driven by reduced temporal correlation to primary sensorimotor cortices. The comparison of XNKQ acupuncture with the other two (non-stimulation) interventions showed no significant differences in EC. Our findings support the importance of the stimulation component of the acupuncture intervention and hint toward the modulation of functional connectivity by XNKQ acupuncture, especially in areas involved in motor function. As a next step, similar mechanisms should be validated in stroke patients suffering from motor deficits.

**ClinicalTrials.gov ID:** NCT02453906

## Background

The acupuncture procedure consists of at least two components: stimulation and point location (Nierhaus et al., 2016; Langevin and Wayne, 2018). According to Chinese Medicine (TCM) theory, different types of stimulation will provide different clinical effects. The stimulation of the needle is mostly accompanied by a needle sensation that is called “deqi” in Chinese Medicine (Kong et al., 2007; Pach et al., 2011). Stimulation that elicited deqi was shown in a PET study to increase blood flow in the hypothalamus, insula, and subcortical structures compared with minimal or non-stimulation after needle insertion (Hsieh et al., 2001). Another study showed that acupuncture impacted selective attention networks, enhancing the efficiency of the alerting and executive control networks, and that acupuncture had a significantly greater effect on the alerting network compared to painful stimulation (Liu et al., 2013). Therefore, the subjective quality and the intensity of the stimulation seem to have an impact on the brain activity changes observed (Hui et al., 2005, 2009; Huang et al., 2012).

The role of location or point specificity in acupuncture is still controversial (Choi et al., 2012; Langevin and Wayne, 2018) and might depend on whether this role is evaluated (i) from the perspective of acupuncture with its concept of meridians and extra points or (ii) from the perspective of modern anatomy and physiology, which can take into account dermal, muscular, and neural components as well as connective tissue and chemical aspects (Nierhaus et al., 2016). In a previous trial of our group, point-specific cerebral responses were shown for one acupuncture point (ST36) in comparison to two control locations (Nierhaus et al., 2015b; Long et al., 2016).

Numerous neuroimaging studies that evaluated the impact of acupuncture on the brain hinted toward specific brain activity and functional connectivity changes due to acupuncture (Dhond et al., 2007; Huang et al., 2012; Chae et al., 2013). Most of these studies either investigate manual acupuncture only on one single point or investigate the effects of electro-acupuncture (Huang et al., 2012). The latter applies an electrical current between acupuncture needles inserted into the skin and is therefore easier to evaluate due to its better potential for blinding, standardization, and easier simultaneous multipoint application. However, in most clinical settings (in China and the West), manual needle-stimulation on multiple acupuncture points is applied.

Xingnao Kaiqiao (XNKQ) acupuncture is a semi-standardized manual acupuncture technique developed in Tianjin, China, by Professor Shi Xuemin using a specific set of acupuncture points and strong needle-stimulation for different neuropathological conditions such as acute and chronic stroke symptoms (Shi, 2013) and multiple sclerosis. In a clinical setting, it was shown that stroke patients suffering from motor deficits react especially well to XNKQ acupuncture (杜蓉 et al., 2015). This suggests an impact of XNKQ on central mechanisms. However, modulation of brain activity following XNKQ acupuncture has not yet been fully investigated.

In the present study, we aimed to evaluate the impact of XNKQ acupuncture (and its components “stimulation” and “point location”) on resting-state functional MRI connectivity. For this, we developed a 2 × 2 design that varied the stimulation component (stimulation vs. non-stimulation) and the location component of acupuncture (acupuncture points vs. non-acupuncture points). We applied data-driven eigenvector centrality mapping (ECM) to evaluate functional connectivity differences for the stimulation component (comparison of stimulated and non-stimulated acupuncture conditions) as well as the point location component (comparison of conditions with acupuncture points and non-acupuncture points).

We had assumed that it would be possible to detect a difference between stimulated and non-stimulated acupuncture conditions as well as between acupuncture conditions which used the established acupuncture point location according to Chinese medicine and conditions which used non-acupuncture point locations. Moreover, we hypothesized that XNKQ acupuncture differed from the three control conditions.

## Materials and Methods

### Subjects

We studied 24 healthy volunteers between 18 and 36 years of age (26.1 ± 4.3 years *(SD)*; 12 females). They gave written informed consent to participate in the experiment according to the declaration of Helsinki. The ethics committee of Charité - Universitätsmedizin Berlin approved the study (Ethics No EA1/338/14) and the study was registered (ClinicalTrials.gov NCT02453906).

Prior to participation, all volunteers underwent a clinical neurological examination and they confirmed they were not taking any medications for acute or chronic diseases.

According to the Edinburgh inventory for the assessment of handedness (Oldfield, 1971), all subjects were right-handed [laterality score: 90.1 ± 17.3 (SD) over a range of −100 (fully left-handed) and +100 (fully right-handed)].

### Design

In a 2 × 2 factorial within-subject design we evaluated four different interventions in four different sessions (four different days with at least 24 h in between each) in a random order: (a) XNKQ acupuncture as manual needle-stimulation on five acupuncture points (DU26 unilaterally, PC6 and SP6 bilaterally); (b) insertion of needles on the five XNKQ acupuncture points without stimulation; (c) manual needle-stimulation on five nearby non-acupuncture points; and (d) insertion of needles on five non-acupuncture points without stimulation. The intervention had a duration of 5 min and was applied in the MRI scanner room. Resting-state fMRI was acquired before and after each intervention to compare changes (post minus pre) in functional connectivity between interventions. The break between pre- and post-resting state scans was about 10 min. Before informed consent, subjects were informed that they would receive acupuncture with five needles at the lower leg, the forearm, and above the upper lip on four separate days, on 2 days with stimulation of the needle and on the other two without. Subjects were blinded regarding the point specificity (acupuncture points vs. non-acupuncture points).

### Point Locations

**Figure 1:**
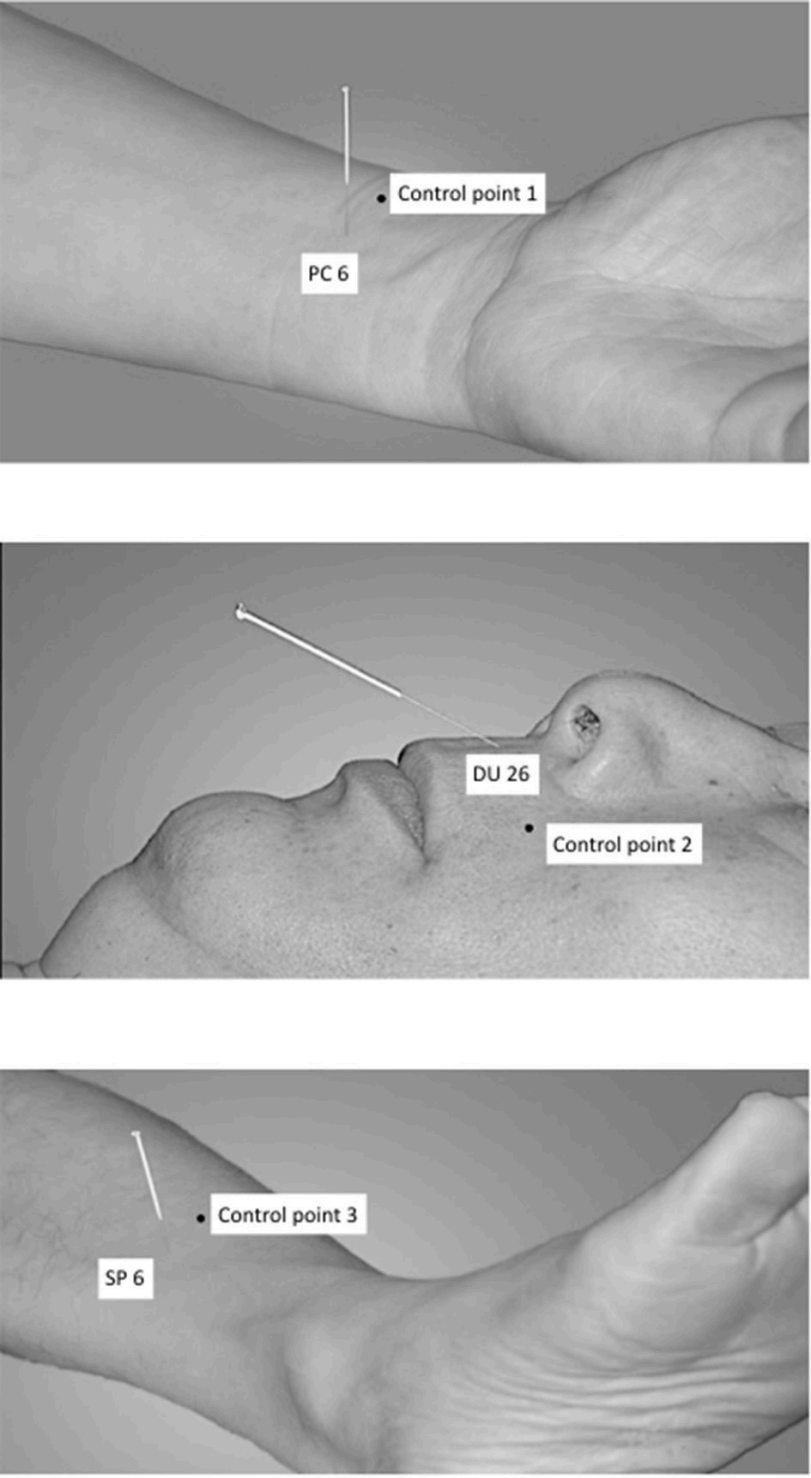
Acupuncture points and respective control points.

The following acupuncture points have been used: PC6 (nei guan, bilateral), DU26 (ren zhong, unilateral), and SP6 (san yin jiao, bilateral) (Shi, 2002, 2013), see Figure 1.

DU26 is located at the junction of the upper 1/3 and middle 1/3 of the philtrum. PC6 is located 2 cun above the transverse crease of the wrist, between the tendons of radial wrist flexor and palmaris longus. SP6 is located 3 cun above the tip of the medial malleolus, behind the posterior border of the medial aspect of the tibia (Shi, 2002).

The control points have been used in an earlier study on XNKQ acupuncture (李筱媛 and 李军, 2009) and were chosen after a discussion process with acupuncture experts. They are not located on a meridian or above a main nerve and are within a radius of 2.5 cm of the respective acupuncture point (Figure 1). Control point 1 (control of PC 6) is located laterally to PC 6, between the lung meridian of hand-taiyin and the pericardium meridian of hand-jueyin (李筱媛 and 李军, 2009). Control point 2 (control of DU 26) is located on the vertical line of the mouth, left-horizontal to DU26 (李筱媛 and 李军, 2009). Control point 3 (control of SP 6) is located six cun above the tip of the medial malleolus, between the spleen meridian of foot-taiyin and the liver meridian of foot-jueyin. It is three cun above the tip of the medial malleolus, in front of the inner of the tibia, 1.25 cun in front of SP 6 (李筱媛 and 李军, 2009).

### Acupuncture Procedures

The acupuncture was performed while the subjects lay in a supine position on the scanner bed by an acupuncturist from Tianjin University of Traditional Chinese Medicine (YC) trained for 12 years and with 8 years of clinical experience. The acupuncturist was trained directly by the developer of the XNKQ acupuncture and had long-time experience in the application of XNKQ acupuncture in patients in China and was also familiar with German study settings. For the acupuncture, sterile, single use, individually wrapped acupuncture needles (0.20 × 30 mm; titanium, DongBang, Acupuncture, Inc., Boryeong, Korea) were used.

For the XNKQ acupuncture PC6 was punctured bilaterally to a depth of 0.5–1.0 cun and stimulated with the reducing method by lifting and thrusting with simultaneous twirling manipulation for 1 min (twirling anticlockwise with the left hand and clockwise with the right hand). After this, DU26 was punctured obliquely toward the nasal septum to a depth of ~0.3–0.5 cun with bird-pecking needling until the eyes became wet or developed tears. Subsequently, SP6 was punctured on both sides obliquely along with the medial border of the tibia to a depth of ~0.5–1.0 cun, with lifting and thrusting reinforcing manipulation, thrusts with heavy strength and lifting with gentle strength for 1 min. The needles were removed directly after stimulation [called “quick needles” technique (Shi, 2013)].

Control condition 1 consisted of the insertion of needles on the same five acupuncture points in the same order as used for XNKQ acupuncture (PC6 bilaterally, DU26 unilaterally, SP6 bilaterally), but without needle-stimulation.

Control condition 2 consisted of manual needle-stimulation on the five nearby non-acupuncture points (control point 1 bilaterally, control point 2 unilaterally, control point 3 bilaterally), identically to the XNKQ needle-stimulation.

Control condition 3 consisted of the insertion of needles on the five non-acupuncture points (control point 1 bilaterally, control point 2 unilaterally, control point 3 bilaterally) without manual needle-stimulation.

Needle sensation as a proxy for deqi and pain sensation was measured after each session by the Massachusetts General Hospital Acupuncture Sensation Scale [MASS, (Kong et al., 2007)].

### MRI Data Acquisition

Before all measurements, participants were instructed to keep the eyes open and to stay relaxed. Data was acquired using a 3T Tim Trio Siemens MRI System (Siemens Medical, Erlangen, Germany) equipped with a 12-channel head coil. For resting-state fMRI images, we used a T2^*^-weighted echo planar imaging (EPI) sequence (37 axial slices, in-plane resolution is 3 × 3 mm, slice thickness = 3 mm, flip angle = 70°, gap = 0.3 mm, repetition time = 2,000 ms, echo time = 30 ms). A structural image was acquired for each participant using a T1-weighted MPRAGE sequence (repetition time = 1,900 ms, inversion time = 900 ms, echo time = 2.52 ms, and flip angle = 9, voxel size 1 × 1 × 1 mm). Subjects' heads were immobilized by cushioned supports, and they wore earplugs to protect against MRI gradient noise throughout the experiment.

### Resting-State fMRI Data Analysis

We removed the first ten volumes of each resting-state scan (RS_pre and RS_post for each subject and all four interventions) to account for adaptation of the participant to scanner noise and environment. We performed slice time correction, head motion correction, and spatial normalization to MNI152 space with SPM12 (www.fil.ion.ucl.ac.uk/spm/). The toolbox REST (www.restfmri.net) was used for temporal band-pass filtering (0.01–0.08 Hz). We did not regress out the global mean signal since this step might affect the correlation between time courses (Buckner et al., 2009; Lohmann et al., 2010; Fransson et al., 2011; Taubert et al., 2011). The anatomical T1-images were normalized to MNI152 space and then segmented into gray matter, whiter matter and cerebral spinal fluid (CSF). Average masks were generated for gray matter, white matter, and CSF derived from the segmented T1 images of all subjects. Principal component analysis (CompCor) was done by the DPABI toolbox (toolbox for Data Processing & Analysis of Brain Imaging, http://becs.aalto.fi/~eglerean/bramila.html) within the CSF/white matter mask on the resting-state data (Behzadi et al., 2007). The first five principal components and six head motion parameters were used as nuisance signals to regress out associated variance. We did not apply spatial smoothing before the centrality analysis, as this could generate artificially high correlation coefficients (Zuo et al., 2012).

To compare differences in head motion across resting-state scans, we calculated the frame-wise displacement (FD) using BRAMILA tools (Power et al., 2012) (http://becs.aalto.fi/~eglerean/bramila.html). The average FD for all scans was examined with two two-factorial ANOVAs, including (a) the factors “session” (1–4) and “time” (pre-post) and (b) the factors “condition” and “time.”

We used the data-driven ECM approach to characterize whole-brain functional connectivity without prior assumptions (Nierhaus et al., 2015a; Long et al., 2016; Antonenko et al., 2018). This graph theoretical network approach quantifies the correlation of each voxel with all other voxels in the brain, aiming to identify how “central” (or prominent) this region is within the whole-brain network (Lohmann et al., 2010). For each individual resting-state scan, the EC map has been generated within the gray matter mask by using fastECM, which provides a more efficient way to perform the centrality analysis without calculating the voxel-wise correlation matrix (Wink et al., 2012). Z-standard transformation (i.e., for each voxel, subtract the mean value of the whole brain then divide by the standard deviation of the whole brain) and 6 mm FWHM smoothing was performed on the individual ECM maps (Zuo et al., 2012; Yan et al., 2013). To evaluate the impact of the four different acupuncture conditions on EC, we analyzed the difference EC-maps (post minus pre) in a 2 × 2 flexible factorial design [correlated repeated measures (Gläscher and Gitelman, 2008)] with the factors “stimulation” (stimulation vs. non-stimulation of the needles) and “location” (acupuncture points vs. non-acupuncture points) within SPM12 with age, gender, and MASS index as covariate. For statistical analysis, we determined cluster-extent thresholds with Monte Carlo simulation (AlphaSim procedure) as implemented in Neuroelf Version 1.1 (http://neuroelf.net) using a family-wise error (FWE) cluster level correction of pFWE < 0.05.

The ECM analysis identifies brain regions with altered overall (whole-brain) connectivity, however it does not show to which specific brain areas the connectivity has changed. A complementary seed-based functional connectivity analysis can be applied to characterize the “origin” of observed EC changes.

The comparison of XNKQ with stimulation on non-acupuncture points revealed EC changes in subthalamic brain regions which are known to be involved in motor control, such as the subthalamic nucleus and red nucleus. In order to show that this result might be connected to cortical motor areas, we have performed a complementary seed-based analysis. As two examples, we chose the red nucleus and subthalamic nucleus as seed regions: For all resting-state scans, we calculated the temporal correlation between the time series of the seed region and all other voxels within the gray matter mask. The difference in the resulting correlation maps were analyzed in a 2 × 2 flexible factorial design with the factors “stimulation” and “location” with age, gender, and MASS index as covariate (similar to the ECM analysis). It should be noted that no formal statistics was applied in this step (double dipping), rather it was used to identify the most affected connections, which contributed most to the statistically significant effect of the ECM analysis. For visualization of the most prominent clusters, we used voxel-wise whole brain FWE correction with peak-level *p* < 0.05.

## Results

### Head Motion

There was no significant difference in head motion (mean FD) across all resting-state scans (4 pre- and 4 post-acupuncture scans) for either the comparison between the four session or the comparison between the four acupuncture conditions (all *p* > 0.46). Over all 8 scans, the mean FD was 0.14 ± 0.06 mm [mean ± std] and the average percentage of volumes exceeding an FD-threshold of 0.5 mm was 1.9 ± 2.8% [mean ± std].

### Needle Stimulation Decreases Eigenvector Centrality in Precuneus

**Figure 2:**
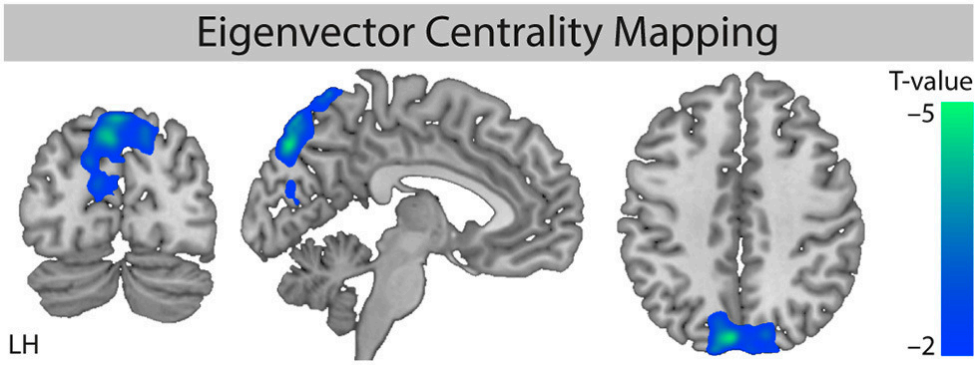
Comparison of ECM values for the factor “stimulation.” Whole brain analysis for the contrast [stimulation vs. non-stimulation] shows decreased centrality for the stimulation conditions. Monte Carlo simulation with AlphaSim was used to identify significant clusters at pFWE < 0.05 (family-wise error correction for multiple comparisons). MNI slice coordinates *X* = −5, *Y* = −78, *Z* = 42. LH, left hemisphere.

For the factor “stimulation,” we found a significant cluster of decreased eigenvector centrality (stimulation < non-stimulation, Figure 2) in the parietal lobe mainly including the precuneus (maximum T value −4.01, left hemisphere (LH), MNI-coordinates *X* = −3, *y* = −78, *Z* = 54) and the cuneus (Tmax = −4.68, LH, *X* = −6, *y* = −81, *Z* = 42).

Further evaluation of the four different conditions showed that the decreased eigenvector centrality in the precuneus was driven by the stimulation of non-acupuncture points (non-points with stimulation, npws). It was significant for the stimulation of non-acupuncture points compared to the two non-stimulation conditions (npws vs. points non-stimulation, pns and npws vs. non-points non-stimulation, npns), and it was not significant when XNKQ was compared to the two non-stimulation conditions (XNKQ vs. pns and XNKQ vs. npns). However, the comparison of the two stimulation conditions (XNKQ vs. npws) also showed no significant difference in precuneus.

The factor location showed no statistically significant different eigenvector centrality. Additionally, no effect was found for the comparison of the non-stimulation conditions (pns vs. npns).

### XNKQ Modulates Functional Connectivity of Motor Areas

**Figure 3:**
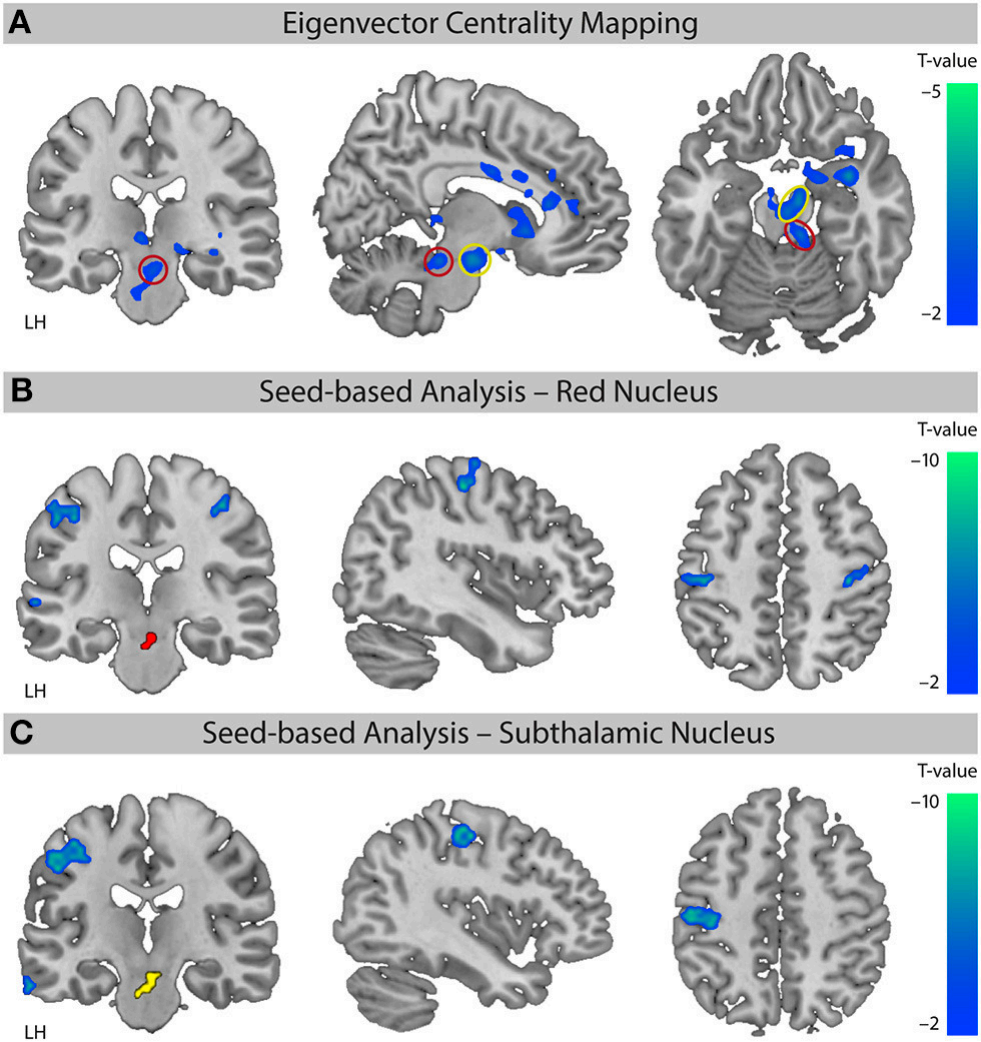
Comparison of the two stimulation conditions. Effect on functional connectivity for the contrast [XNKQ vs. “non-acupuncture point with stimulation” (npws)]. **(A)** Data-driven ECM analysis shows decreased centrality for XNKQ, mainly in subcortical regions. Monte Carlo simulation with AlphaSim was used to identify significant clusters at pFWE < 0.05 (family-wise error (FWE) correction for multiple comparisons). Red circle (Red Nucleus) and yellow circle (Subthalamic Nucleus) indicate areas that were used for seed-based correlation analyses. MNI slice coordinates *X* = **–**8, *Y* = **–**22, *Z* = **–**18. **(B)** Seed-based functional connectivity with seed in Red Nucleus. Whole brain analysis shows for XNKQ decreased temporal correlation of the seed-region (red) with primary sensorimotor areas (voxel-wise FWE correction at pFWE < 0.05). MNI slice coordinates *X* = 41, *Y* = **–**22, *Z* = 51. **(C)** Seed-based functional connectivity with seed in Subthalamic Nucleus. Whole brain analysis shows for XNKQ decreased temporal correlation of the seed-region (yellow) with left primary sensorimotor areas (voxel-wise FWE correction at pFWE < 0.05). MNI slice coordinates *X* = **–**40, *Y* = **–**20, *Z* = 48. LH, left hemisphere.

The EC analysis for XNKQ compared with the stimulation of non-acupuncture points (XNKQ vs. npws) showed one significant cluster (mainly subcortical) of decreased centrality (Figure 3A). This cluster included subcortical structures such as the caudate (right hemisphere (RH), Tmax = −3.71, MNI-coordinates *X* = 12, *y* = 15, *Z* = −3), the subthalamic nucleus (RH: Tmax = −3.57, *X* = 9, *y* = −12, *Z* = −18), the thalamus (LH: Tmax = −3.48, *X* = −6, *y* = −27, *Z* = −3; RH: Tmax = −3.42, *X* = 15, *y* = −24, *Z* = −6), the lentiform nucleus (RH, Tmax = −3.80, *X* = 27, *y* = 3, *Z* = −12), and the red nucleus (RH, Tmax = −3.13, *X* = 9, *y* = −30, *Z* = −18), as well as the claustrum (RH, Tmax = −5.06, *X* = 36, *y* = 0, *Z* = −15), the anterior cingulate cortex (RH, Tmax = −2.94, *X* = 12, *y* = 36, *Z* = 9), and the cingulate cortex (RH, Tmax = −2.74, *X* = 9, *y* = −3, *Z* = 30). No significant effect was found for XNKQ compared to the two non-stimulation conditions (XNKQ vs. pns and XNKQ vs. npns).

The complementary seed-based functional connectivity analysis revealed that the decreased EC in subthalamic areas was mainly driven by decreased temporal correlation of the respective seed region to primary sensorimotor areas (S1/M1); the red nucleus showed significantly decreased correlation mainly to bilateral S1/M1 (RH: Tmax = −6.00, *X* = 42, *y* = −19, *Z* = 55; LH: Tmax = −6.05, *X* = −51, *y* = −22, *Z* = 46; Figure 3B) and the subthalamic nucleus mainly to left S1/M1 (LH: Tmax = −7.04, *X* = −39, *y* = −22, *Z* = 49; Figure 3C).

### Needle Sensation

Needle sensation was measured for all four conditions using the MASS index: XNKQ mean 4.17 95%CI[3.34;5.01]; npws 3.44[2.54;4.34]; pns 3.52[2.65;4.39]; and npns 2.29[1.58;3.00]. The statistical analysis showed a significant main effect for both factors (stimulation and location) in our 2 × 2 factorial design (stimulation vs. non-stimulation conditions p = 0.030; acupuncture points vs. non-acupuncture points *p* = 0.018). The comparison of the four conditions showed significant differences only for npns comparisons (XNKQ vs. NPNS, *p* = 0.001; NPWS vs. NPNS *p* = 0.043; PNS vs. NPNS *p* = 0.028).

## Discussion

To elucidate cerebral effects of manual acupuncture using more than one acupuncture point, we applied XNKQ acupuncture and three control conditions in a neuroimaging study in healthy subjects. In a 2 × 2 factorial within-subject design, we investigated the impact of the factors stimulation and location, and of XNKQ acupuncture specifically, on resting-state functional connectivity.

While the factor location appears to have no significant effect on centrality, we found decreased eigenvector centrality in the precuneus for the factor stimulation. This result was driven by the stimulation of non-acupuncture points, as the comparison of XNKQ acupuncture with the two non-stimulation interventions showed no significant differences. However, when comparing XNKQ acupuncture with manual needle-stimulation on non-acupuncture points, we found significantly decreased functional connectivity for areas involved in motor function.

Our results support the assumption that (1) needle-stimulation drives the cerebral effects, (2) point location only impacts connectivity when the acupuncture points are stimulated, and (3) XNKQ acupuncture, as a complex form of acupuncture, modulates functional connectivity in motor areas minutes after the acupuncture.

Our study design shows strengths and weaknesses that should be considered when interpreting the results. With our factorial within-subject design including a relatively large number of healthy subjects measured on four separate days, we were able to separate the factors stimulation and location, as well as to reduce variance and carry over effects. The design we chose aimed at a study question relevant for the understanding of clinical acupuncture which primarily uses manual needle-stimulation on more than one point. So far, numerous imaging studies evaluate only one-point acupuncture and/or apply electro-acupuncture (Huang et al., 2012; Chae et al., 2013). Such a setting might be better suited for standardization and blinding options but does not represent clinical acupuncture in usual care settings.

Although in our study design only five locations were acupunctured for a relatively short time interval (hence still does not fully represent the clinical setting), we were able to observe cerebral changes that illustrate the impact of a complex acupuncture, such as XNKQ acupuncture, on the brain. Because the duration of the sustained effect of acupuncture is not known, we do not know whether pausing the intervention for at least 24 h is sufficient to avoid carry-over effects. However, the order of interventions was randomized to minimize the risk of a systematic impact.

We chose a design that can evaluate rapid effects on resting-state functional connectivity, which are observable after the intervention, but not the instant evoked responses of the different acupuncture conditions. This design decision may decrease the sensitivity to identify differences between the conditions. For the evaluation of instant effects, an event-related design with needling during the scanning phase would have been necessary. However, this would be much more difficult to achieve, especially when evaluating manually stimulated acupuncture on multiple acupuncture points.

Although we included a relatively large number of subjects, the sample size might be too low to show robust effects. The level of statistical significance we chose was liberal. For future studies with a similar design, an even larger sample size might be recommended, especially for the evaluation of effects in patients. Based on our findings, it is now possible to evaluate a hypothesis-driven approach, which might create more robust results in contrast to the data-driven approach we chose as the primary analysis.

In our study, we included only healthy subjects for an easy-to-standardize setting to understand the neurophysiology of the different acupuncture conditions. However, usually XNKQ acupuncture is only applied in a clinical setting for patients with neurological deficits such as multiple sclerosis or stroke as part of a multi-component intervention that also includes physiotherapy. Therefore, it is possible that effects in healthy subjects differ from effects expected in patients. However, a study on patients would have created more variance and is more prone to bias, which is not ideal as a first step.

However, only the subjects were blinded for the applied acupuncture conditions as well as the researchers analyzing the data during the first stages of analyses. The acupuncturist applying the manual acupuncture could not be blinded for the different conditions and this might have had an effect on needle-stimulation. However, we measured needle sensation as a proxy for stimulation strength and included it into our statistical model.

The choice of control points for an acupuncture study is very challenging because it is still not clear what constitutes an acupuncture point, and it is difficult to combine the traditional concept of acupuncture with modern anatomy (Nierhaus et al., 2016; Langevin and Wayne, 2018). Therefore, it is possible that the control points chosen for our study were not inert, either from the perspective of acupuncture or from the perspective of anatomy.

To our surprise, we found no significant differences between XNKQ and the two non-stimulated acupuncture conditions. This means that the main effect that we found in precuneus for the factor “stimulation” is driven by the needle-stimulation on non-acupuncture points. However, the comparison within the two stimulated acupuncture conditions (XNKQ vs. stimulation on non-acupuncture points) revealed a significant difference—mainly in subcortical regions—that is not observed in the other comparisons. It seems that the stimulation of acupuncture points (XNKQ) induces subcortical connectivity changes (decreased centrality) that are opposite to the connectivity changes induced by needle-stimulation of “neutral” non-acupuncture points. This result supports the view that both “stimulation” and “point location” contribute to the acupuncture effect.

Other studies have also shown that acupuncture can affect functional connectivity of brain networks such as the default mode network (DMN) or sensorimotor network in pain, stroke, or mental conditions (Dhond et al., 2008; Bai et al., 2009; Hui et al., 2009; Chae et al., 2013; Napadow et al., 2013; Liang et al., 2014; Li et al., 2014; Zhao et al., 2014; Deng et al., 2016). Numerous studies could show that the precuneus (as part of the DMN) is frequently affected by acupuncture (Chae et al., 2013; Nierhaus et al., 2015b). So far, the specific role of the precuneus is not fully understood, however for pain it might be involved in the assessment and integration of pain (Goffaux et al., 2014). The reduced centrality that we found for the precuneus in resting-state after needle-stimulation might hint toward such cerebral processing induced by the strong and (sometimes) painful stimulation.

Functional connectivity is regularly affected by stroke (Grefkes and Fink, 2011; Rehme and Grefkes, 2013; Baldassarre et al., 2016; Almeida et al., 2017), and brain imaging studies have revealed functional brain reorganization in relation to recovery (Schaechter, 2004; Almeida et al., 2017). In a stroke mouse model, it could be shown that multisensory input can improve functional recovery and resting-state functional connectivity after stroke (Hakon et al., 2018). Acupuncture can be regarded as a complex somatosensory input with needle-stimulation. According to a study by Li et al. both acupuncture and somatosensory stimuli to the contralesional side produce hyperactivation in the ipsilesional primary sensorimotor cortex and SII (Dhond et al., 2007). A study by Schaechter et al. (2007) revealed that after acupuncture intervention (verum or sham), patients exhibited changes in motor cortex activity associated with the stroke-affected hand that were positively correlated with changes in somatosensory-motor function of the affected upper limb. There was a trend toward greater increases in motor cortex activity in patients treated with verum acupuncture than sham acupuncture (Dhond et al., 2007). XNKQ is an acupuncture technique specially designed for different neuropathological conditions such as acute and chronic stroke symptoms (Shi, 2013), and moreover seems to impact on patients suffering from motor deficits. Therefore, our results are well in line with the existing literature and support the assumption that XNKQ affects the motor system.

Our data-driven analysis (ECM) showed that XNKQ acupuncture affects functional connectivity of subcortical areas (e.g., red nucleus or subthalamic nucleus) that are known to be involved in motor function (Milardi et al., 2016). This is supported by our complementary seed-based analysis, which showed reduced functional connectivity between the seed regions and primary sensori-motor areas after XNKQ acupuncture. Maybe this reduced functional connectivity in the motor systems allows for a better reorganization during recovery from motor deficits in stroke. Of course, it needs to be proven if this can be translated to stroke patients.

## Conclusion

Our findings support the importance of the stimulation component of the acupuncture intervention and hint toward the modulation of functional connectivity by XNKQ acupuncture, especially in areas involved in motor function. As a next step, similar mechanisms should be validated in stroke patients suffering from motor deficits.

## Data Availability

The datasets generated for this study are available on request to the corresponding author.

## Author Contributions

TN, YC, CW, and DP conceived and designed the experiments. TN, YC, BL, and DP performed the trial. TN and DP analyzed the data. TN, DP, YC, and CW wrote the first draft of the paper. TN, YC, BL, XS, MY, CW, and DP discussed the data, revised the paper, and approved the final version.

### Conflict of Interest Statement

The authors declare that the research was conducted in the absence of any commercial or financial relationships that could be construed as a potential conflict of interest.
